# Comprehensive Analysis of Ferroptosis Regulators With Regard to PD-L1 and Immune Infiltration in Clear Cell Renal Cell Carcinoma

**DOI:** 10.3389/fcell.2021.676142

**Published:** 2021-07-05

**Authors:** Song Wang, Shiming Chen, Yufan Ying, Xueyou Ma, Haixiang Shen, Jiangfeng Li, Xiao Wang, Yiwei Lin, Ben Liu, Xiangyi Zheng, Liping Xie

**Affiliations:** Department of Urology, The First Affiliated Hospital, School of Medicine, Zhejiang University, Hangzhou, China

**Keywords:** ferroptosis, clear cell renal cell carcinoma, PD-L1, immune infiltration, cars, therapeutic target

## Abstract

Clear cell renal cell carcinoma (ccRCC) is one of the tumor types with sensitivity to ferroptosis, and immunotherapy has emerged as a standard pillar for metastatic ccRCC treatment, while it remains largely obscure whether ferroptosis influences the tumor immune microenvironment in ccRCC. Based on available data in The Cancer Genome Atlas, divergent expression profiles of ferroptosis regulators were noted in ccRCC and normal tissues, and we also found that the ferroptosis regulators correlated with the PD-L1 expression. Two independent subtypes were determined by consensus clustering analysis according to the expression level of ferroptosis regulators in ccRCC. Cluster 1 showed lower histological tumor stage and grade, more favorable prognosis, and higher PD-L1 expression compared to cluster 2. CIBERSORT analysis revealed that cluster 2 harbored higher infiltrated levels of CD8+ T cell, Tregs, and T follicular helper cell, while cluster 1 more correlated with the monocyte, M1 macrophage, and M2 macrophage. Gene set enrichment analysis indicated that the ERBB signaling and JAK_STAT signaling pathways were significantly enriched in cluster 1. We subsequently identified CARS as the potentially key immune infiltration-related ferroptosis regulator, whose high expression showed dismal prognosis and was positively correlated with PD-L1 expression in ccRCC. We also verified the upregulation of CARS in ccRCC tissues and cell lines *via* qRT-PCR method. Additionally, a pan-cancer analysis demonstrated that CARS closely related to the expression of immune checkpoint-related genes (especially PD-L1) and an unfavorable prognosis in diverse cancer types. In summary, our study suggested the crucial role of ferroptosis in immune infiltration of ccRCC, and CARS is a potentially novel prognostic biomarker and potential target for cancer immunotherapy.

## Introduction

Kidney cancer is a prevalent human malignancy and among the top 10 causes of cancer-related deaths globally, with estimated 430,000 new cases and 180,000 deaths annually (Sung et al., 2021). Clear cell renal cell carcinoma (ccRCC, also named KIRC) is the major subtype, accounting for nearly 70% of kidney cancer cases (Capitanio et al., 2019). Although the improved understanding and advances in cancer treatment are acknowledged, the clinical outcomes of patients with ccRCC remain unsatisfactory, specifically those diagnosed with advanced ccRCC. In recent years, immunotherapy has demonstrated unprecedented antitumor activity in patients with advanced malignancies—emerging as a standard therapeutic modality for recurrent or metastasized ccRCC (Barata and Rini, 2017). Majority of patients with ccRCC are still unable to acquire durable clinical benefits from mainstream immunotherapies, such as the combined therapies of anti-PD-1 and/or anti-CTLA4 antibodies, despite having historically high response rates to these immunotherapies (Motzer et al., 2015; Choueiri et al., 2018). An in-depth understanding of the heterogeneity of ccRCC tumor immune microenvironment (TIME) might help in providing personalized immunotherapy management to improve patient survival.

Ferroptosis is an iron-dependent programmed cell death process, characterized by the production of free radicals and excessive accumulation of lipid peroxides (Dixon et al., 2012). Notably, ferroptosis has been implicated in ischemic organ injuries (Tuo et al., 2017), neurodegeneration (Chen et al., 2015), autoimmune diseases (Hu et al., 2019), and cancer development (Viswanathan et al., 2017). Specifically, significant evidence suggests that ferroptosis plays a vital role in cancer inhibition, and its activation enhances the therapeutic efficacy of anticancer agents (Eling et al., 2015; Yu et al., 2017; Zhang et al., 2021). Nonetheless, most studies have focused on elucidating the intrinsic carcinogenic mechanisms of tumors; whether ferroptosis influences the TIME remains largely elusive. Emerging evidence indicates that tumor-killing T cell and anti-PD-L1 antibodies induce ferroptosis in tumor cells, while liproxstatin-1 (a type of highly efficient ferroptosis depressor) inhibits the anticancer efficacy of these agents. Additionally, a synergistic effect has been reported between anti-PD-L1 antibodies and ferroptosis activators (such as erastin), suppressing tumor growth both *in vitro* and *in vivo* (Wang et al., 2019). However, there is still a lack of integrated understanding of ferroptosis in ccRCC, including the crosstalk between ferroptosis regulators and tumor immune microenvironment.

Here, a systematical analysis was performed, involving the expression profiles and the correlations with prognosis, PD-L1, and roles in TIME of ferroptosis regulators in ccRCC. Besides this, we clustered subtypes based on the expression level of ferroptosis regulators. Consequently, apparent tumor heterogeneity, distinct PD-L1 expression, and tumor immune microenvironment were noted between the two subtypes, promoting risk stratification and precision therapy for patients with ccRCC. Subsequently, CARS was identified as a potential immune infiltration-related ferroptosis regulator, whose high expression demonstrated dismal prognosis and positively correlated with PD-L1 expression in ccRCC. Furthermore, this work also comprehensively explored the pan-cancer expression profiles, prognostic values, and correlations with immune checkpoints of CARS. Our findings provide novel insights into the regulatory mechanisms associated with TIME and the approaches for ccRCC immunotherapy.

## Materials and Methods

### Data Acquisition

RNA-seq data and clinical information of the ccRCC cohort were downloaded from the Genomic Data Commons (GDC) data portal of The Cancer Genome Atlas (TCGA) database (https://portal.gdc.cancer.gov/) in January 2020. Data included 72 normal kidney and 530 ccRCC tissues. In the part of pan-cancer analysis of CARS, RNA-seq data and corresponding clinical information of 33 cancer types cohort were obtained from the GDC portal of TCGA. Meanwhile, normal tissue samples were obtained from GTEx V8 release version (https://gtexportal.org/home/datasets). GSE53757 and GSE15641 datasets from Gene Expression Omnibus (GEO; https://www.ncbi.nlm.nih.gov/geo/) database were downloaded and used to further validate the expression level of CARS. The abbreviations of multiple cancer types, ferroptosis regulators, and immune-related checkpoints in this study are listed in Supplementary Table 1.

### Bioinformatics Analysis

The R software “Consensus Cluster Plus” (v1.54.0) package was adopted for consistency analysis (the maximum number of clusters is six, and 80% of the total sample drawn 100 times) according to the expression level of selected ferroptosis regulators in ccRCC patients; the “ggplot2” package was used for principal component analysis (PCA). The “Cluster Profiler” package was used to perform the gene set enrichment analysis (GSEA), Gene Ontology (GO) analysis, and Kyoto Encyclopedia of Genes and Genomes (KEGG) signaling pathway analysis in R software. The permutation tests were performed 1,000 times, and *p*-values were adjusted for multiple testing by performing the Benjamini–Hochberg procedure, Notably, *p* < 0.05 or FDR < 0.05 was a significantly enriched pathway.

To obtain a reliable evaluation of immune infiltration in ccRCC, CIBERSORT was used to detect the relative proportion of infiltrating immune cells in each ccRCC cancer sample. The algorithm of 1,000 permutations was adopted to obtain meaningful results, and the results were implemented by R, version 4.0.3, and software packages “ggplot2” and “pheatmap.” The protein expression level of CARS in ccRCC compared to normal tissue was obtained from the Human Protein Atlas database (https://www.proteinatlas.org/). The CARS survival was externally validated by using the Kaplan–Meier plotter (Nagy et al., 2018) (http://kmplot.com/analysis/). Univariate and multivariate Cox regression analyses were performed to identify the prognostic value of CARS in ccRCC. Forest was used to show the *P* value, hazard ratio (HR), and 95% CI of each variable *via* the “forestplot” R package.

### Human Clinical Samples, Cell Lines, and qRT-PCR Analysis

In total, 22 pairs of ccRCC and adjacent normal tissues were obtained from patients subjected to radical nephrectomy between January and October 2013 at the First Affiliated Hospital of Zhejiang University. Written informed consent was acquired from each patient, and the Institutional Ethics Committee in the hospital approved this study. The human ccRCC cell lines 786-O, ACHN, and caki-1 and the normal kidney cell line HK-2 were purchased from Shanghai Institute of Cell Biology (Shanghai, China). Total RNA was extracted with TRIzol reagent (Takara) and then reverse-transcribed into cDNA by using PrimeScript RT Reagent Kit (Takara). The CARS relative expression was detected by RT-qPCR, through using the SYBR Premix Ex Taq (Takara) and ABI 7500 fast real-time PCR System (Applied Biosystems). GAPDH was used as an endogenous normalization reference. All patients' clinical information and primers used are shown in Supplementary Tables 2, 3.

### Statistical Analysis

Statistical tests were conducted using R, version 4.0.3, and GraphPad Prism 8.0. Student's *t*-test was used to analyze CARS expression in 22 pairs of ccRCC and adjacent normal tissues; one-way ANOVA was utilized to compare the expression of CARS in ccRCC cell lines to that in normal kidney cell line. Wilcox test and Kruskal–Wallis test were used to perform the group comparisons of two groups and more than two groups, respectively. Spearman correlation analysis was performed to evaluate the correlation between ferroptosis regulators' expression levels with checkpoint -elated genes. For the Kaplan–Meier curves, P-values, and HR with 95% confidence interval (CI) were generated by log-rank tests and univariate Cox proportional hazards regression. Univariate and multivariate analyses were conducted using Cox regression models to establish the independent prognostic value of the CARS integrated other clinical features. The predictive efficiency of genes for 1-, 3-, and 5-year overall survival (OS) was estimated using the receiver operating characteristic (ROC) curves by timeROC package. *P* < 0.05 indicated statistical significance.

## Results

### Expression Divergence of Ferroptosis Regulators Between ccRCC and Adjacent Normal Tissues

**Figure 1:**
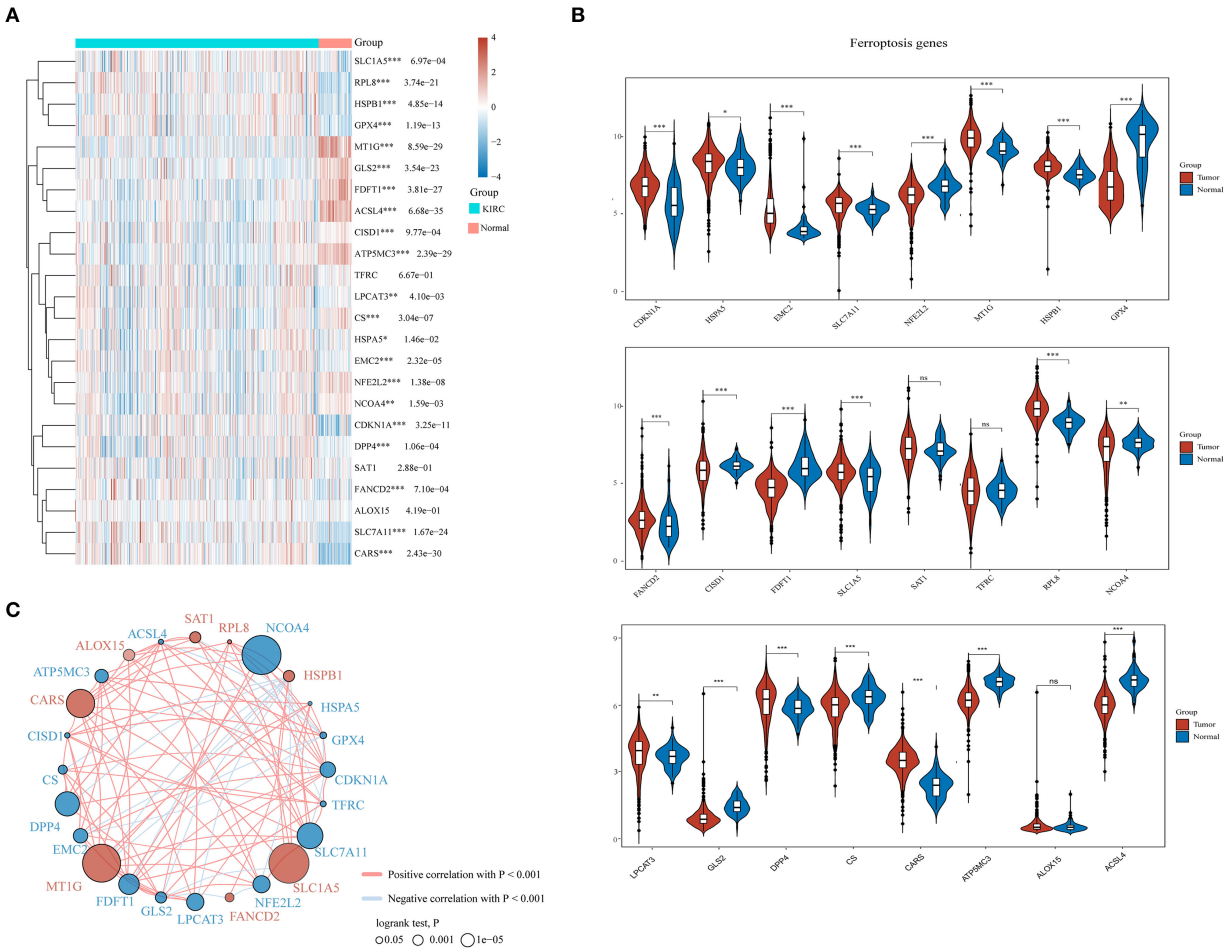
The expression distribution, correlation, and prognostic values of ferroptosis regulators in clear cell renal cell carcinoma (ccRCC) patients. Heat map **(A)** and violin plots **(B)** of ferroptosis regulators in ccRCC compared to normal tissues. **(C)** Spearman correlation and prognostic values of ferroptosis regulators in ccRCC. The red and blue line respectively represents the positive and negative correlation. The red and blue dot represents bad and good prognosis, respectively. The larger the circle, the smaller the prognosis log rank *p*. *p < 0.05, **p < 0.01, and ***p < 0.001.

A total of 24 ferroptosis regulators derived from a previous study were defined as key ferroptosis regulator genes because of their pivotal roles in regulating ferroptosis (Liu et al., 2020). To evaluate the biological functions of ferroptosis regulators in ccRCC, the expression patterns of selected ferroptosis regulators between ccRCC and adjacent normal pairs were systematically explored based upon TCGA dataset. We downloaded and analyzed the expression profiles of 530 cases of ccRCC individuals and 72 normal patients. Consequently, distinct expression levels of ferroptosis regulators were found in ccRCC and normal tissues (Figures 1A,B), including upregulated ferroptosis regulators CDKN1A, HSPA5, EMC2, SLC7A11, MT1G, HSPB1, FANCD2, SLC1A5, RPL8, LPCAT3, DPP4, and CARS (*p* < 0.05) and downregulated regulators NFE2L2, GPX4, CISD1, FDFT1, NCOA4, GLS2, CS, ATP5MC3, and ACSL4 (*p* < 0.05). However, no significant differences were noted between the ccRCC and normal tissues regarding the expression of SAT1, TFRC, and ALOX15 (*p* > 0.05). Furthermore, correlation combined with prognosis analysis demonstrated that the expression of most ferroptosis regulatory genes was positively correlated and played a vital prognostic role in ccRCC (Figure 1C). These findings suggest that ferroptosis regulators might play a crucial role in regulating tumorigenesis and ccRCC development.

### Consensus Clustering Analysis of Ferroptosis Regulators Revealed Significant Differences in Baseline Characteristics and Survival Between Two Patient Clusters

**Figure 2:**
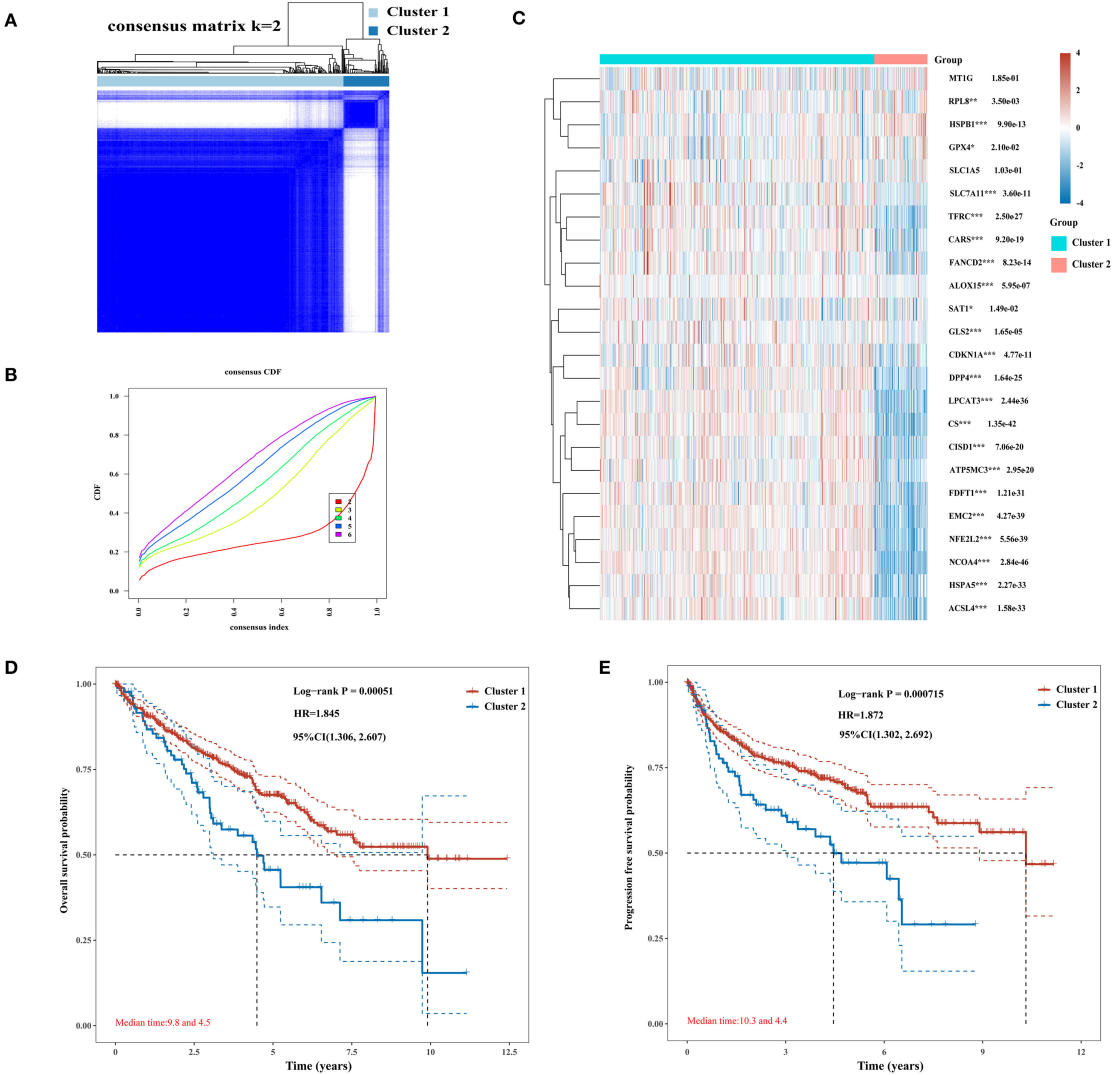
Differential expression pattern of ferroptosis regulators and survival in two clear cell renal cell carcinoma (ccRCC) subtypes. **(A)** Consensus clustering matrix for *k* = 2. **(B)** Cumulative distribution function curves for *k* = 2–6. **(C)** Heat map visualized the expression patterns of ferroptosis regulators in two ccRCC subtypes or clusters. **(D,E)** The Kaplan–Meier curves show the overall survival **(D)** and the disease-free survival **(E)** for two clusters of ccRCC patients. *p < 0.05, **p < 0.01, and ***p < 0.001.

The Consensus Cluster Plus package of R software was used for consistency analysis. According to the expression level of selected ferroptosis regulators and the proportion of ambiguous clustering measure, *k* = 2 was identified as optimal clustering stability from *k* = 2 to 6 (Figures 2A,B; Supplementary Figures 1A–E). Therefore, the 530 KIRC patients were divided into two subtypes, namely, cluster 1 (*n* = 444) and cluster 2 (*n* = 86). In contrast to cluster 2, most of the ferroptosis regulators (20/24) were highly expressed in cluster 1, and two regulators (HSPB1 and GPX4) were lowly expressed in cluster 1, while there was no difference in the expression of two regulators (MT1G and SLC1A5) between two clusters (Figure 2C). Subsequently, differences were noted in the clinicopathological characters and prognosis between the two subtypes, as shown in Table 1. Cluster 1 was preferentially correlated with lower tumor stage as well as cancer grade (*P* < 0.01), whereas no statistical differences in age, sex, and race were found between the two clusters (*P* > 0.05). Additionally, cluster 1 patients possessed favorable OS (*P* < 0.001) and progression-free survival (*P* < 0.001) than cluster 2 (Figures 2D,E). The results indicated that there is significant heterogeneity between the two subtypes of KIRC patients. To further confirm the clustering results defined by the expression of ferroptosis regulators, we subsequently applied the PCA method to analyze the gene expression profiles between the two subtypes (Supplementary Figure 1F). As a result, the gene expression profiles between the two subtypes revealed perfect differences.

**Table 1:** Clinical characteristics of two clusters of ccRCC patients.

	**Feature**	**Cluster 1**	**Cluster 2**	***P*-value**
Age	Mean (SD)	60.4 (12.1)	61.4 (12.6)	
	Median (min, max)	61 (26, 90)	60.5 (33, 85)	0.483
Gender	Female	159	27	
	Male	285	59	0.508
Race	Black or Asian	52	12	
	White	385	74	0.763
pTNM_stage	I	236	29	
	II	48	9	
	III	91	32	
	IV	66	16	0.002
Grade	G1	13	1	
	G2	201	26	
	G3	167	39	
	G4	55	20	0.009

### Association of Ferroptosis Regulators With PD-L1 Expression Level and Immune Cell Infiltration in ccRCC

**Figure 3:**
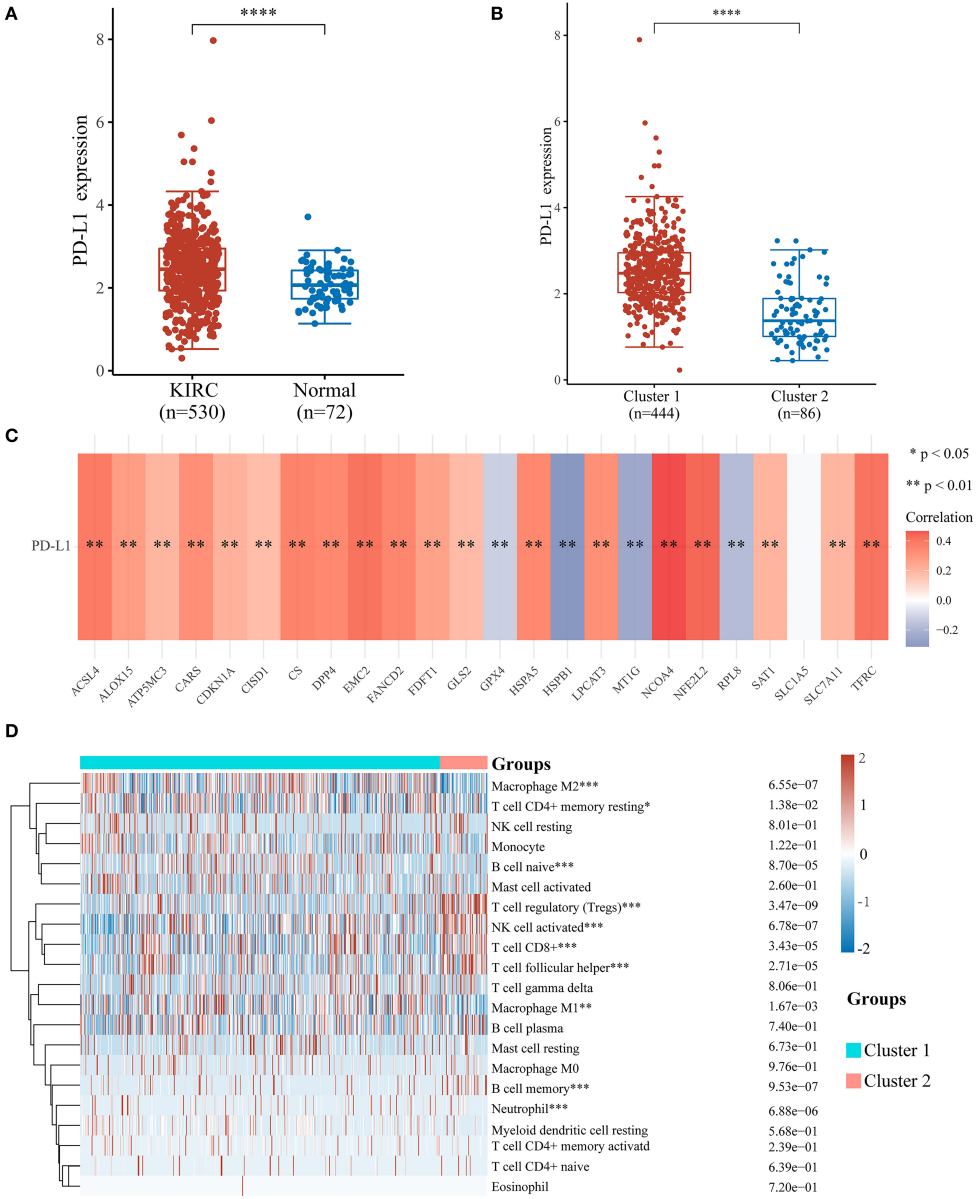
Association of PD-L1 with ferroptosis regulators and the differential infiltration level of tumor immune cells in two clear cell renal cell carcinoma (ccRCC) subtypes. **(A,B)** The expression level of PD-L1 in ccRCC/normal group **(A)** and cluster 1/2 subtype **(B)** in ccRCC. **(C)** The correlation of PD-L1 with ferroptosis regulators in the TCGA-KIRC cohort. **(D)** The infiltrating levels of various immune cell types in two subtypes in the TCGA-KIRC cohort. *p < 0.05, **p < 0.01, ***p < 0.001, and ****p < 0.0001.

**Figure 4:**
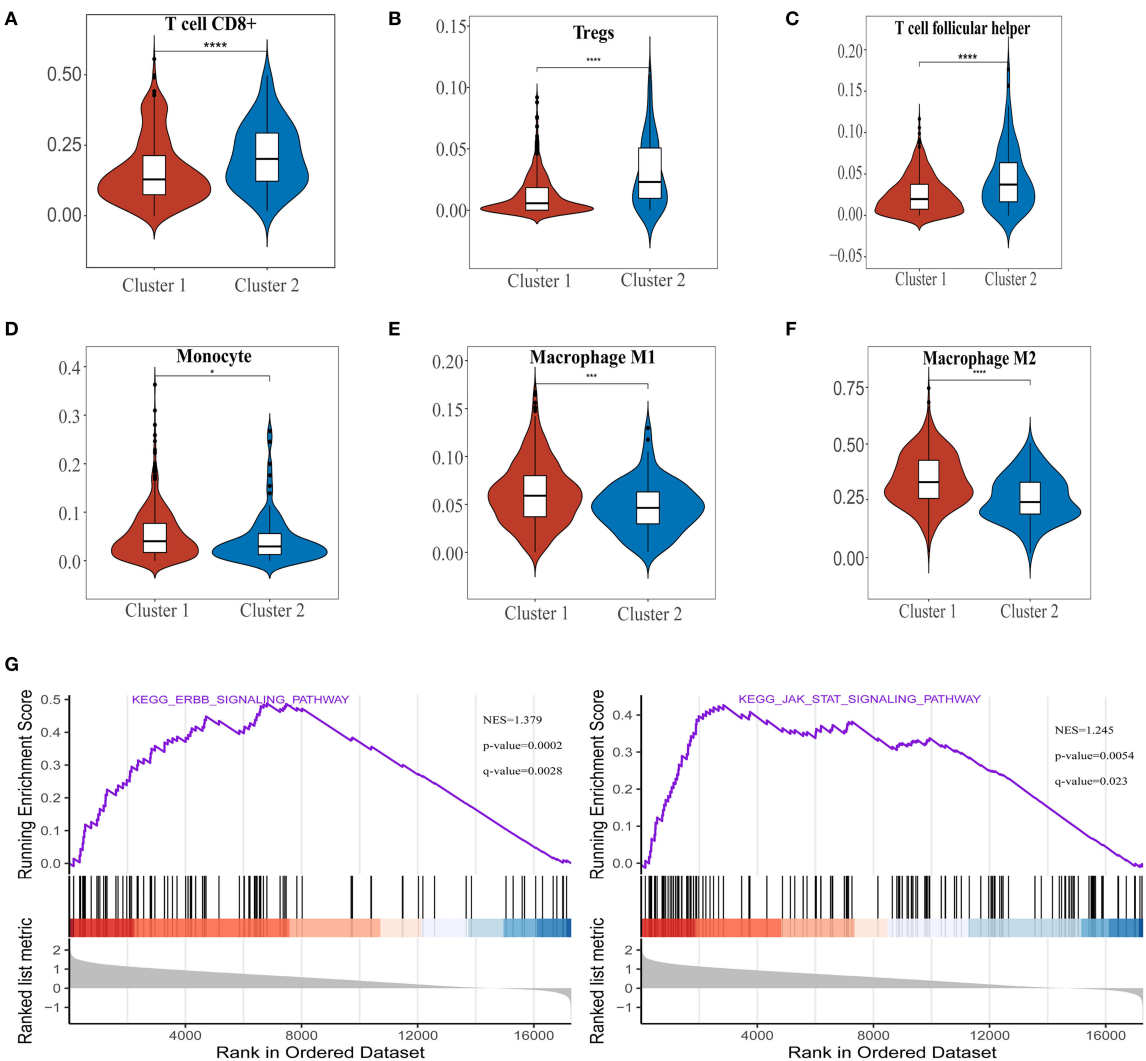
The differences of immune cell infiltration levels in two clear cell renal cell carcinoma (ccRCC) subtypes. **(A–F)** The infiltration level of the CD8+ T cell **(A)**, Tregs **(B)**, and T follicular helper cell **(C)** were downregulated in cluster 1 ccRCC patients. The infiltration levels of the monocyte **(D)**, M1 macrophage cell **(E)**, and M2 macrophage cell **(F)** were upregulated in cluster 1 ccRCC patients. **(G)** Gene set enrichment analysis indicated that ERBB signaling and the JAK-STAT signaling pathways are significantly enriched in cluster 1. NES, normalized enrichment score; *q*-value, false discovery rate. *p < 0.05, ***p < 0.001, and ****p < 0.0001.

To investigate the relationship between PD-L1 and ferroptosis in ccRCC, this work evaluated the divergent expression in two clusters and the association of PD-L1 with the ferroptosis regulators in ccRCC. Unlike normal adjacent tissues and cluster 2 patients, the expression levels of PD-L1 in ccRCC tissues and cluster 1 patients were distinctly overexpressed, respectively (*P* < 0.01; Figures 3A,B). An analysis involving 530 ccRCC individuals revealed that PD-L1 showed a positive association with the expression levels of most ferroptosis regulators (such as ACSL4, CARS, NCOA4, etc.) but a negative association between HSPB1, MT1G, RPL8, GPX4, and PD-L1 (Figure 3C). We further explore the impact of ferroptosis regulators on the tumor immune microenvironment of ccRCC. The two subtypes classified according to the expression levels of selected ferroptosis regulators revealed significant differences in immune cell infiltration (Figure 3D). Cluster 2 possessed higher infiltrated levels of activated NK cell, memory B cell, CD8+ T cell, Tregs, and T follicular helper cell (Figures 3D, 4A–C), while cluster 1 was more correlated with the CD4+ memory resting T cell, naive B cell, neutrophil, monocyte, M1 macrophage, and M2 macrophage (Figures 3D, 4D–F). The GSEA method was adopted to clarify the underlying regulatory mechanisms causing the TIME difference between the two clusters. Consequently, ERBB signaling (normalized enrichment score, NES = 1.379, *p* and *q* < 0.01) and JAK_STAT signaling (NES = 1.245, *p* and *q* < 0.05) were significantly associated with cluster 1 (Figure 4G). Therefore, the ERBB and JAK_STAT signaling pathways are potentially implicated in the divergent TIME of two subtypes in ccRCC.

### Key Ferroptosis Regulator CARS Is Upregulated in ccRCC

**Figure 5:**
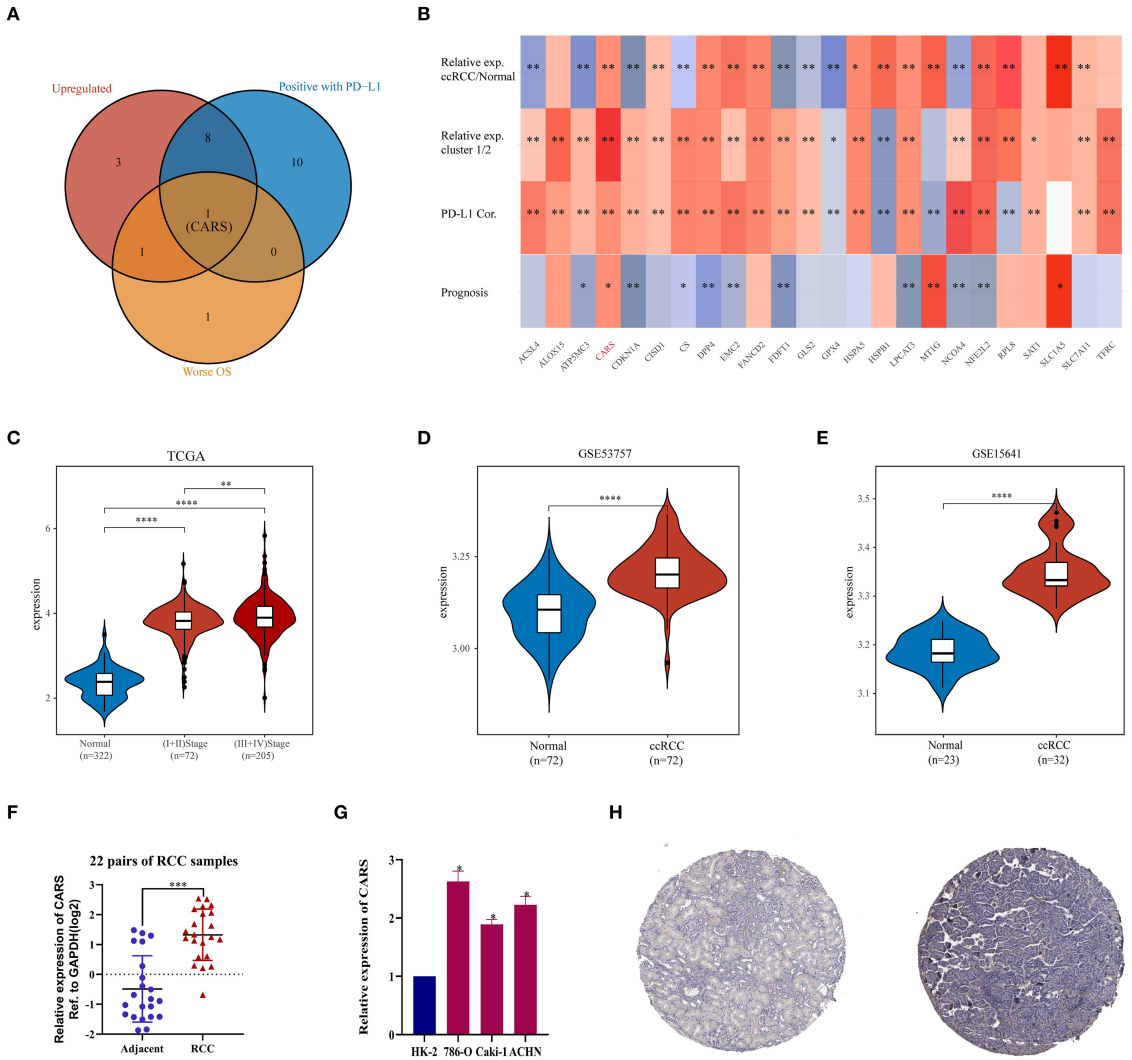
Analysis of the CARS gene expression in ccRCC. **(A)** The Venn diagram suggested that the upregulation of CARS was an unfavorable prognostic factor and positively correlated with the PD-L1 expression in clear cell renal cell carcinoma (ccRCC). **(B)** The heat map visualized the relative expression of 24 ferroptosis regulators in ccRCC/normal (row 1) and cluster 1/2 (row 2) and the correlations of 24 regulators with the PD-L1 expression (row 3) and prognosis (row 4). The red color indicates a high expression in ccRCC or cluster 1, positively correlated with PD-L1 or bad prognosis. *p < 0.05 and **p < 0.01. **(C)** CARS was upregulated in ccRCC tissues compared to normal tissues, and the expression significantly correlated with AJCC stages. **(D,E)** Validation sets showed that CARS was highly expressed in ccRCC tissues compared to adjacent normal tissues in GSE53757 **(D)** and GSE156417 **(E)**. **(F)** Relative expression of CARS was detected by qRT-PCR in 22 pairs of ccRCC and normal tissues. **(G)** Compared with HK-2 cell line (normal), CARS was highly expressed in 786-O, ACHN, and caki-1 cell lines (ccRCC). **(H)** Immunohistochemistry staining indicated that, compared with normal kidney tissue (left), CARS was significantly elevated in ccRCC tissue (right) in the human protein atlas (antibody HPA002384, ×10). *p < 0.05, **p < 0.01, ***p < 0.001, and ****p < 0.0001.

As described previously, the two ccRCC subtypes defined according to the expression levels of ferroptosis regulators have apparent tumor heterogeneity and distinct PD-L1 expression and tumor immune microenvironment. These results suggested that ferroptosis regulators play an important role in tumor development and tumor immune infiltration. An intersection of ferroptosis regulators highly expressed in ccRCC, linked to poor prognosis, and positively correlated with the expression of PD-L1 was used to determine key regulators linked to poor prognosis and PD-L1 expression among the 24 selected ferroptotic regulators. As shown in Figure 5A, CARS was the potentially key poor prognostic ferroptosis regulator that is highly expressed in ccRCC and positively correlated with PD-L1 expression. Additionally, CARS was the only ferroptosis regulator highly expressed in ccRCC and cluster 1, positively corelated with PD-L1 expression and worse OS (Figure 5B). To further illustrate the expression of CARS in ccRCC, we analyzed the expression data from the TCGA and GEO database. The results showed that, in the TCGA cohort and GSE53757 and GSE15641 cohorts, CARS was significantly more highly expressed in ccRCC than in normal tissues (*P* < 0.05, Figures 5C–E). Moreover, the expression level of CARS was significantly higher in advanced cancer stage compared to early-cancer-stage patients (*P* < 0.05, Figure 5C). Subsequently, the relative expression of CARS was verified in 22 pairs of ccRCC and normal tissues in ccRCC cell lines and normal kidney cell line *via* qRT-PCR method (*P* < 0.05, Figures 5F,G). Correspondingly, the protein expression of CARS was also upregulated in ccRCC tissue compared to normal tissue (Figure 5H). These results demonstrated that CARS is highly expressed in ccRCC at both transcriptional and translational levels.

### High CARS Expression Was a Significantly Independent Prognostic Biomarker in ccRCC

**Figure 6:**
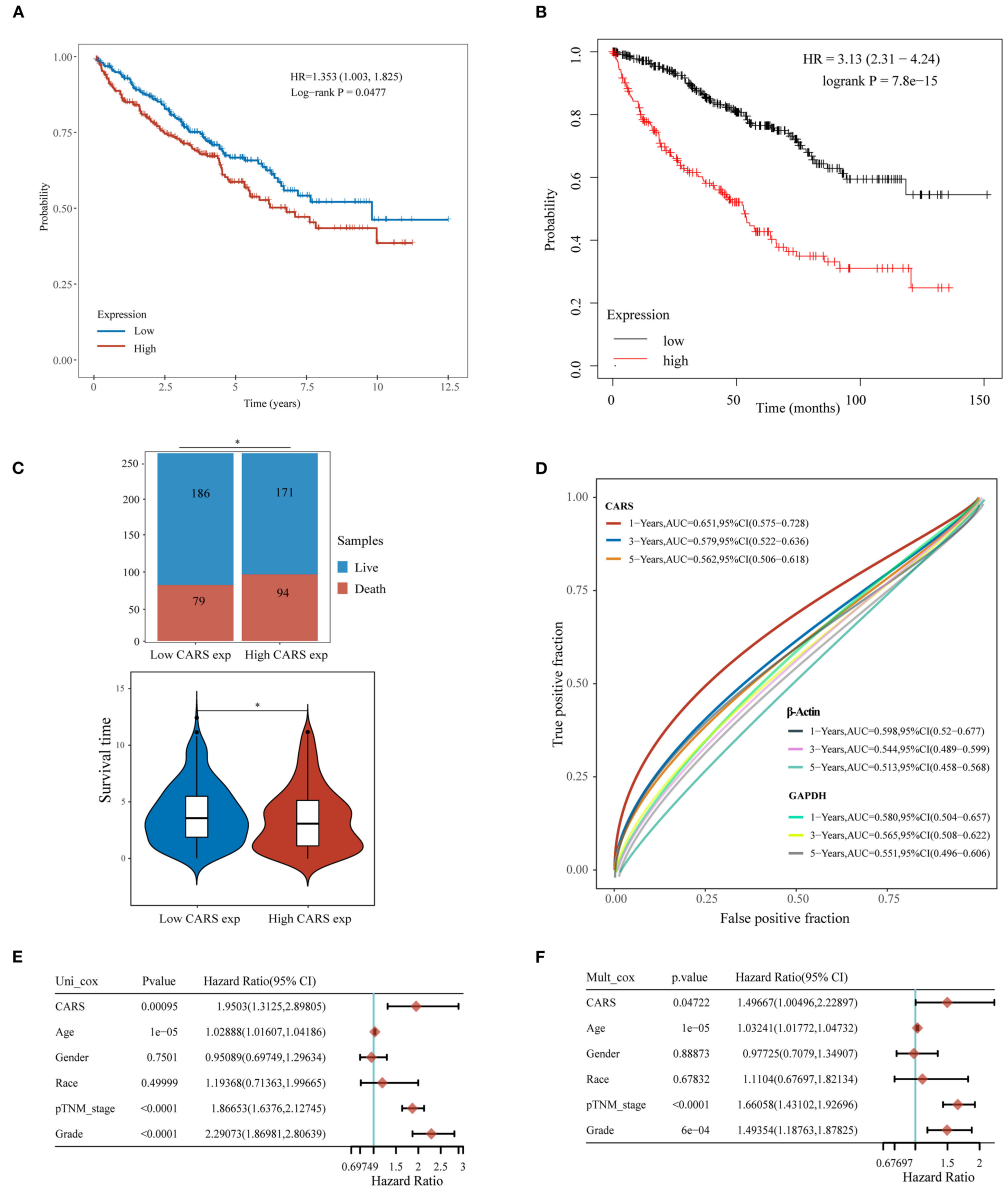
Upregulated CARS expression is associated with poor outcomes of clear cell renal cell carcinoma (ccRCC). **(A,B)** The Kaplan–Meier analysis of ccRCC patients with high and low CARS expression level in the TCGA cohort **(A)** and Kaplan–Meier plotter database **(B)**. **(C)** The stacked bar chart and violin plot visualized the proportion of live/death and survival time in high/low CARS groups in ccRCC patients. **(D)** Time-dependent receiver operating characteristics analysis of CARS and the two most common housekeeping genes, GAPDH and β-Actin. **(E,F)** Forest plots based upon the outcomes of univariate **(E)** and multivariate Cox regression **(F)** of CARS expression and other clinicopathological factors. *p < 0.05.

Furthermore, the prognostic value of highly expressed CARS was analyzed in ccRCC, where the upregulated CARS group show worse OS compared to the lowly expressed group (*P* < 0.05, Figure 6A). A similar result was also confirmed in an independent ccRCC cohort from the Kaplan–Meier plotter (*P* < 0.05; Figure 6B). A stacked bar chart and a violin plot (shown in Figure 6C) demonstrated that high CARS ccRCC patients possessed a lower proportion of live/death and survival time compared to the low CARS ccRCC patients (*P* < 0.05). Besides this, CARS also showed a powerful prognostic ability since the ROC curve showed that the AUC values of CARS expression for predicting 1-, 3-, and 5-year survival were 0.651, 0.579, and 0.562, while the AUC values of β-actin and GAPDH expression for predicting 1-, 3-, and 5-year survival were 0.598, 0.544, and 0.513 and 0.580, 0.565, 0.551, respectively (Figure 6D). The Cox analysis method was also adopted to explore the relationship between CARS expression and OS in ccRCC; univariate analysis revealed that age (HR = 1.0289, *p* = 1e-05), TNM stage (HR = 1.866, *p* < 0.0001), tumor grade (HR = 2.2907, *p* < 0.0001), and CARS expression (HR = 1.9503, *p* = 0.00095) are significantly correlated with OS (Figure 6E). Moreover, a multivariate analysis, exhibited as a forest plot in Figure 6F, revealed that CARS expression (*p* = 0.047) is an independent factor for prognosis in ccRCC patients.

### Correlation Analysis of CARS Expression With PD-L1 and Infiltrating Immune Cells

**Figure 7:**
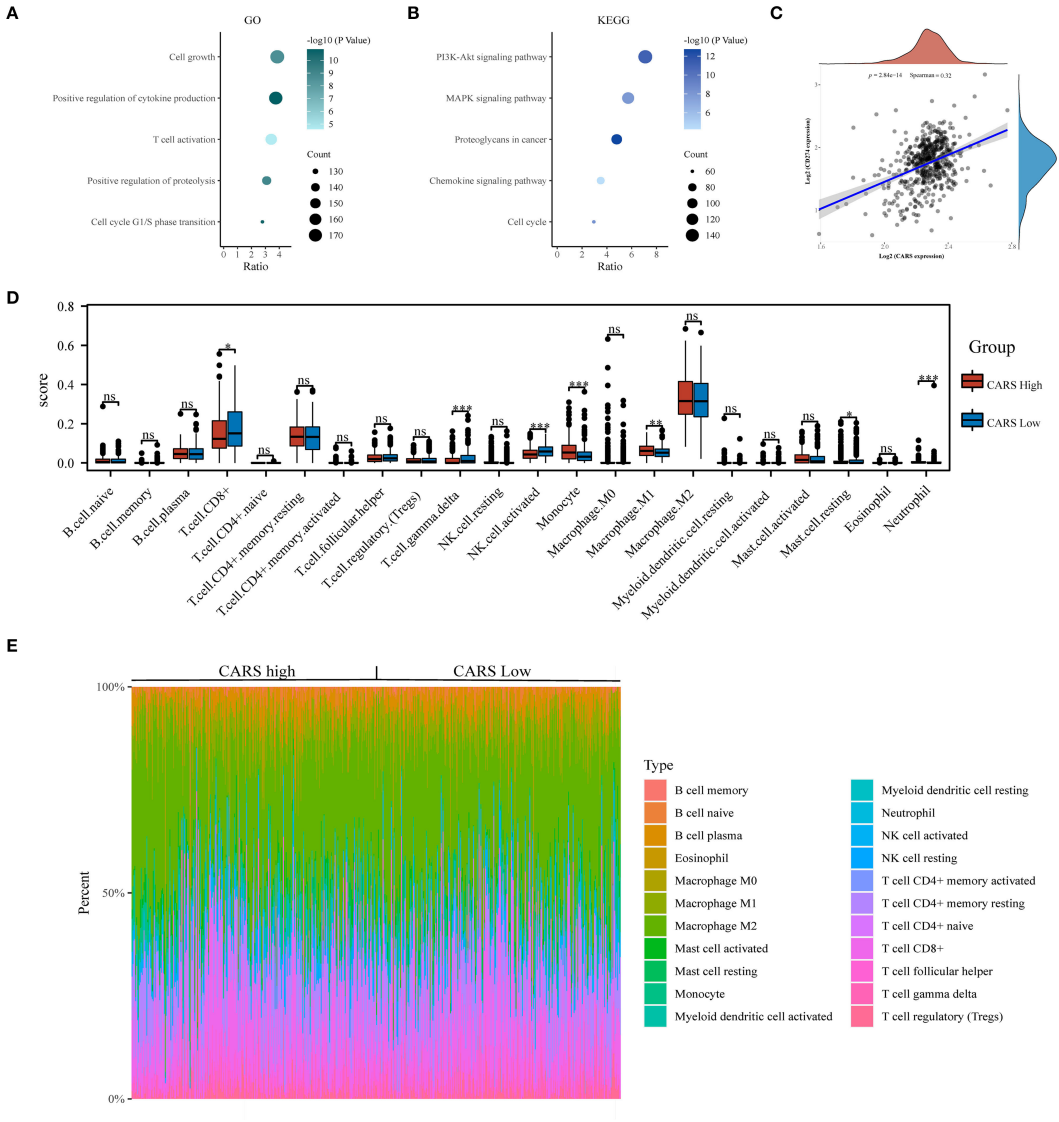
Correlation analysis of CARS expression with PD-L1 and infiltrating immune cells in clear cell renal cell carcinoma (ccRCC). **(A,B)** The enriched Gene Ontology **(A)** and Kyoto Encyclopedia of Genes and Genomes signaling pathways **(B)** analysis of highly expressed CARS in ccRCC. **(C)** Spearman correlation analysis of CARS expression and PD-L1 expression in ccRCC. **(D)** The infiltrating levels of immune cells in high and low CARS expression groups in ccRCC patients. **(E)** The heat map visualized the percentage abundance of tumor-infiltrating immune cells in each sample. *p < 0.05, **p < 0.01, and ***p < 0.001.

To further investigate the underlying functions of CARS in ccRCC, we utilized the Cluster Profiler package to analyze GO term and KEGG pathway in samples with high expression levels of CARS (as shown in Figure 7A). The biological processes of GO annotation suggested five categories that were positively linked to highly expressed CARS: cell growth, positive regulation of cytokine production, T cell activation, positive regulation of proteolysis, and cell cycle G1/S phase transition. KEGG pathway analysis revealed that five pathways positively associated with the expression of CARS: PI3K-AKT signaling pathway, MAPK signaling pathway, proteoglycans in cancer, chemokine signaling pathway, and cell cycle (Figure 7B). These results indicated that the CARS may be involved in multiple signaling pathways and tumor immune microenvironment in ccRCC. A further correlation analysis showed that CARS was significantly positively correlated with the expression of PD-L1 in ccRCC (*P* < 0.001, Spearman = 0.32) (Figure 7C). To identify a discrepancy in the TIME of ccRCC patients with high and low expression levels of CARS, the CIBERSORT algorithm was carried out to explore the difference of numerous immune cell subtypes between the CARS high expression group and the low expression group in ccRCC. As shown in Figure 7D, monocytes, M1 macrophages, and neutrophil increased in the high CARS expression group, whereas CD8+ T cell, activated NK cell, and gamma delta T cell decreased (*P* < 0.05), and the heat map visualized the percentage of abundance of tumor-infiltrating immune cells in each sample of low and high CARS group (Figure 7E).

### Comprehensive Analysis of Key Ferroptosis Regulator CARS in Pan-Cancer

**Figure 8:**
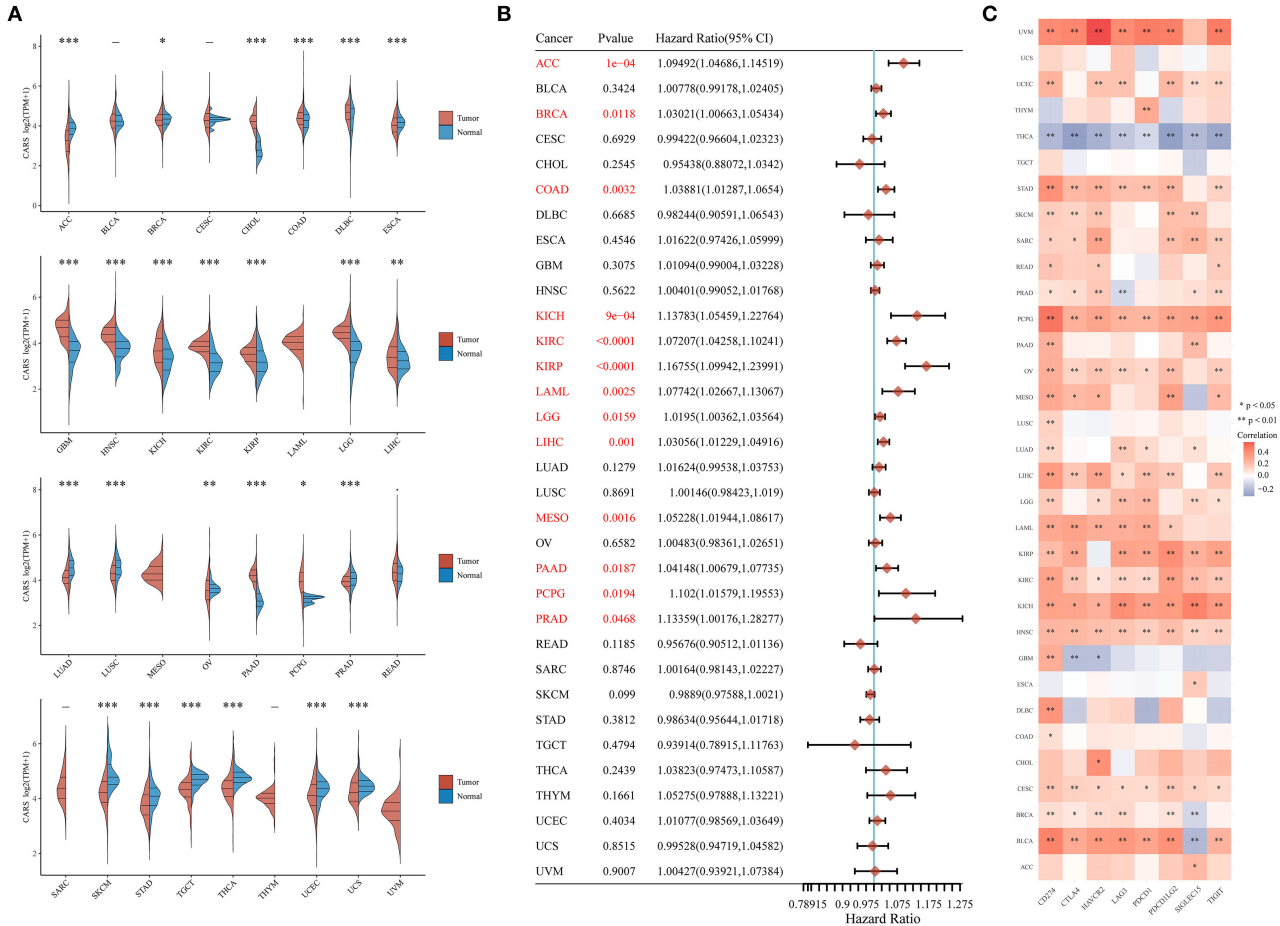
Comprehensive analysis of key ferroptosis regulator CARS in pan-cancers. **(A)** CARS was differentially expressed in most cancer types. **(B)** Highly expressed CARS showed poor overall survival in various cancers. **(C)** The association of CARS expression with the expression of eight common immune checkpoints was observed in most cancer types, especially the expression level of PD-L1. *p < 0.05, **p < 0.01, and ***p < 0.001.

Considering that ferroptosis and immune infiltration are widely implicated in the occurrence and development of multiple cancer types, we desired to determine whether CARS, the key ferroptosis regulator of our study, also predicts poor prognosis and affects the tumor immune microenvironment in other cancer types. Therefore, we firstly analyzed the relative expression of CARS in more than 10,000 tumor and normal tissue samples in the TCGA and GTEx database. The results demonstrated that, compared to normal tissues, CARS was significantly upregulated in 12 (CHOL, COAD, DLBC, GBM, HNSC, KICH, KIRC, KIRP, LGG, LIHC, PAAD, and PCPG) cancer types and downregulated in 13 (ACC, BRCA, ESCA, LUAD, LUSC, OV, PRAD, SKCM, STAD, TGCT, THCA, UCEC, and UCS) cancer types (*P* < 0.05; Figure 8A). Meanwhile, the prognostic values of CARS were also investigated across multiple cancer types. The univariate Cox regression analyses in pan-cancer suggested that the upregulation of CARS was correlated with poor OS in 13 (ACC, BRCA, COAD, KICH, KIRC, KIRP, LAML, LGG, LIHC, MESO, PAAD, PCPG, and PRAD) cancer types (*P* < 0.05, Figure 8B). In addition, a further expression correlation analysis between CARS and eight most common immune checkpoint-related genes (including PD-L1, CTLA4, HAVCR2, LAG3, PDCD1, PDCD1LG2, SIGLEC15, and TIGIT) was performed. The results revealed that CARS closely related to the expression of checkpoint-related genes in most cancer types, especially the expression of PD-L1 (Figure 8C). In summary, a high expression of CARS was correlated with immune checkpoint-related gene expression and dismal prognosis in diverse prevailing cancers.

## Discussion

Ferroptosis is a novel iron-dependent programmed cell death process, which is characteristically distinct from autophagy, apoptosis, and other forms of necrosis (Lei et al., 2020). Recently, emerging evidence reported the vital roles of ferroptosis in the tumorigenesis of human malignancies (Ding et al., 2021), especially the ccRCC which is intrinsically vulnerable to ferroptosis (Zou et al., 2019). However, the potential role of ferroptosis in the ccRCC immune microenvironment remains elusive. Here we distinguished two independent subtypes with differential clinical features, prognoses, PD-L1 expression, and TIME *via* consensus clustering for selected ferroptosis regulators. Among these, CARS was identified as the potential immune infiltration-related ferroptosis regulator. Furthermore, pan-cancer analysis revealed a close correlation of CARS with the expression of PD-L1 and poor overall survival in multiple cancer types.

In recent years, precision medicine has revolutionized the treatment of human cancers (Dupont et al., 2021), which emphasizes that, by integrating multidimensional biological data and clinical characteristics, highly heterogeneous cancers can be subdivided into more accurate subtypes for individualized treatment (Zhang et al., 2020). Indeed accumulating evidence established subgroups of tumor patients according to their molecular profiles, representing distinct phenotypes, prognoses, and therapy responses (Yan et al., 2018; Tan et al., 2019). For instance, Zhou et al. classified colorectal cancer patients into high- and low-risk groups according to their autophagy features, and consequently, more aggressive and targeted therapies were required in the high-risk group (Zhou et al., 2019). Based on immune-related gene expression signatures, lung adenocarcinoma patients can be divided into two prognostically and clinically relevant subtypes. Patients from the high-risk immune subtype were more responsive to immune checkpoint blockade treatment (Wang et al., 2020). In this study, we applied consensus clustering analysis to classify ccRCC patients into two subgroups or clusters on the basis of ferroptosis regulator expression signatures. Low tumor grade, favorable prognosis, and upregulated PD-L1 expression level were observed in cluster 1 patients. This also meant that cluster 1 patients benefit more from anti-PD-L1 antibodies even with the occurrence of metastasis. Meanwhile, further analysis revealed that cluster 1 harbored significantly higher infiltrated levels of monocyte, M1 macrophage cell, and M2 macrophage cell. On the other hand, cluster 2, with unfavorable survival, was more correlated with CD8+ T cell, Tregs, and T follicular helper cells. The role of Tregs and T follicular helper cells, which were upregulated in cluster 2, in tumor immune escape was well-established (Serrels et al., 2015). Intriguingly, CD8+ T cells are a type of well-known effector cells. Unlike the vast majority of cancers, high CD8+ T cell infiltration level was recognized as an adverse prognostic factor in ccRCC (Braun et al., 2020; Bruni et al., 2020; Dai et al., 2021). Moreover, Clark et al. classified ccRCC into four subtypes. Among these, CD8+-inflamed ccRCC with a highly infiltrated level of CD8+ T cell is tightly linked to an increased proportion of higher-grade tumors and worse prognosis, while CD8- inflamed tumors with low infiltration of CD8+ T cell were characterized by an innate immune signature, which showed an increased level of macrophages (Clark et al., 2019). Additionally, several recent studies suggested that tumor-killing T cell and anti-PD-L1 antibodies can induce ferroptosis in tumor cells, while liproxstatin-1 (ferroptosis depressor) can reduce the anticancer efficacy of these agents (Lang et al., 2019; Wang et al., 2019). These findings suggest an overwhelming complexity in the role of ferroptosis in the TIME. To further explore the possible mechanisms of the distinct TIME between two clusters, we carried out the GSEA analysis. The results revealed that the ERBB signaling and the JAK_STAT signaling were significantly enriched in cluster 1. Notably, ERBB family members activated downstream of signaling pathways are widespread, including the PI3K–AKT–mTOR and MAPK pathways, which not only participate in the endogenous carcinogenic pathway but also affect the tumor immune microenvironment (Yarden and Pines, 2012). Yi et al. disclosed that activation of PI3K–AKT–mTOR signaling suppresses ferroptosis in cancer (Yi et al., 2020). Liu et al. found elsewhere that the interaction between GPX4 and mTOR signaling modulates ferroptotic cancer cell death (Liu et al., 2021). Ferroptosis regulator DPP4 was reported to activate the MAPK pathway in papillary thyroid carcinoma (Hu et al., 2021). These studies have indicated that the interaction between ferroptosis and ERBB signaling pathway might be implicated in regulating the TIME of ccRCC.

Given the vital roles of ferroptosis in ccRCC TIME, we identified adverse prognostic regulators among the selected 24 ferroptosis regulators, elevated in tumors and positively correlated with PD-L1 expression. Interestingly, CARS was the only key ferroptosis regulator corresponding to these three conditions. CARS (also CARS1) promote cysteine metabolism (Akaike et al., 2017), hence regulating ferroptosis. Hayano et al. demonstrated that the knockdown of CARS can induce the transsulfuration pathway, causing cystine deprivation and thereby inhibiting ferroptosis in osteosarcoma, Ewing's sarcoma, and pancreatic carcinoma (Hayano et al., 2016). A recent study found that CARS1 has a unique domain (UNE-C1) that influences the immune response (Cho et al., 2020). Nevertheless, the potential role of CARS in ccRCC TIME remains puzzling. Herein we demonstrated the overexpression and unfavorable prognostic value of CARS in multiple ways. Furthermore, CIBERSORT analysis revealed that the levels of monocyte and M1 macrophages were increased in the high CARS expression group, whereas the levels of CD8+ T cell and NK-activated cells were reduced. This implied a possible regulation role of CARS in the polarization of TAM. Notably, significantly high infiltration levels of NK-activated cells were observed in the low CARS expression group. NK-activated cells are a type of cytotoxic cell; they are directly cytotoxic to tumor cells but also produce cytokines (TNF-α, IFN-γ, etc.), inhibiting cancer angiogenesis and proliferation (Whiteside, 2020). Therefore, CARS might play a pivotal role in the regulation of the tumor microenvironment in ccRCC. Given that ferroptosis is widely involved in various biological processes, we sought to establish whether CARS, the key ferroptosis regulator of our study, also plays a vital role in other cancer types. Subsequently, a pan-cancer analysis was performed, and the results demonstrated that CARS was closely related to the expression of immune checkpoint genes (specifically the expression of PD-L1) in most cancer types. Also, CARS overexpression showed poor OS in 13 cancer types but showed no protective effect in any cancer types. These results implied that CARS is a potential target for cancer immunotherapy and an effective prognostic biomarker not limited to ccRCC.

Nevertheless, several potential limitations in this study deserve to be mentioned. First, we selected 24 key ferroptosis regulators for analysis in this study, according to a comprehensive previous study. Thus, it is probable that ferroptosis regulators were left out before and after our analysis. Furthermore, the clustering subtypes as well as the interactions between the TIME and ferroptosis regulators were analyzed only based on available data from the TCGA due to lacking sufficient data in our own cohort. As such, further reliable validation analysis in larger cohorts needs to be carried out in the future. Besides this, additional experiments *in vitro* and *in vivo* are warranted to be performed to elucidate the regulatory mechanism between ferroptosis regulators and TIME.

In summary, this research systematically analyzed the expression profiles and the correlations with prognosis, PD-L1, and role in TIME of ferroptosis regulators in ccRCC. The ferroptosis regulators' expressions were significantly associated with PD-L1. Two independent subtypes were also established by consensus clustering for ferroptosis regulators. Obvious tumor heterogeneity, distinct PD-L1 expression, and TIME were observed between the two subtypes, which will contribute to the risk stratification and precision therapy for patients with ccRCC, and we also found that the interaction between ferroptosis and the ERBB signaling pathway might be implicated in regulating the TIME of ccRCC. Among the selected ferroptosis regulators, CARS was subsequently identified as an independent prognostic indicator of ccRCC patients and positively correlated with PD-L1 expression. Further pan-cancer analysis demonstrated that CARS closely related to the expression of immune checkpoint genes (especially the expression of PD-L1) and poor prognosis in diverse cancer types. Certainly, a series of clinical trials and basic researches might help to determine targets for enhancing the efficacy of cancer immunotherapy.

## Data Availability Statement

The datasets presented in this study can be found in online repositories. The names of the repository/repositories and accession number(s) can be found in the article/Supplementary Material.

## Ethics Statement

The studies involving human participants were reviewed and approved by First Affiliated Hospital, School of Medicine, Zhejiang University. The patients/participants provided their written informed consent to participate in this study. Written informed consent was obtained from the individual(s) for the publication of any potentially identifiable images or data included in this article.

## Author Contributions

SW drafted and wrote the manuscript. SW, XM, YY, and HS collected related data. SW and SC conducted the bioinformatics analysis with R software. JL, YL, and BL searched the existing related literatures. XW, XZ, and LX revised the manuscript. All the authors read and approved the final manuscript.

## Conflict of Interest

The authors declare that the research was conducted in the absence of any commercial or financial relationships that could be construed as a potential conflict of interest.

## Supplementary Material

Supplementary Figure 1 Consensus clustering for ferroptosis regulators in clear cell renal cell carcinoma (ccRCC). **(A–D)** Four heat maps exhibit the clustering matrix for ferroptosis regulators in ccRCC patients for *k* = 3, 4, 5, and 6. The tighter and clearer the clusters are, the more optimal the cluster. **(E)** Delta area curve of consensus clustering for *k* = 2–6. **(F)** Principal component analysis of ccRCC patients' ferroptosis regulator expression profiles demonstrates two patient clusters.

Supplementary Table 1 The abbreviations of multiple cancer types, ferroptosis regulators and immune-related checkpoints in this study.

Supplementary Table 2 The patients' clinical information (*n* = 22) in this study.

Supplementary Table 3 The oligonucleotides used in this study. ^*^F, forward primer; R, reverse primer.
