# Supplementary figures and images for: Comprehensive Analysis of Ferroptosis Regulators With Regard to PD-L1 and Immune Infiltration in Clear Cell Renal Cell Carcinoma
Source: Front Cell Dev Biol. 2021 Jul 5;9:676142. doi: 10.3389/fcell.2021.676142 (PMC8287329; doi:10.3389/fcell.2021.676142)

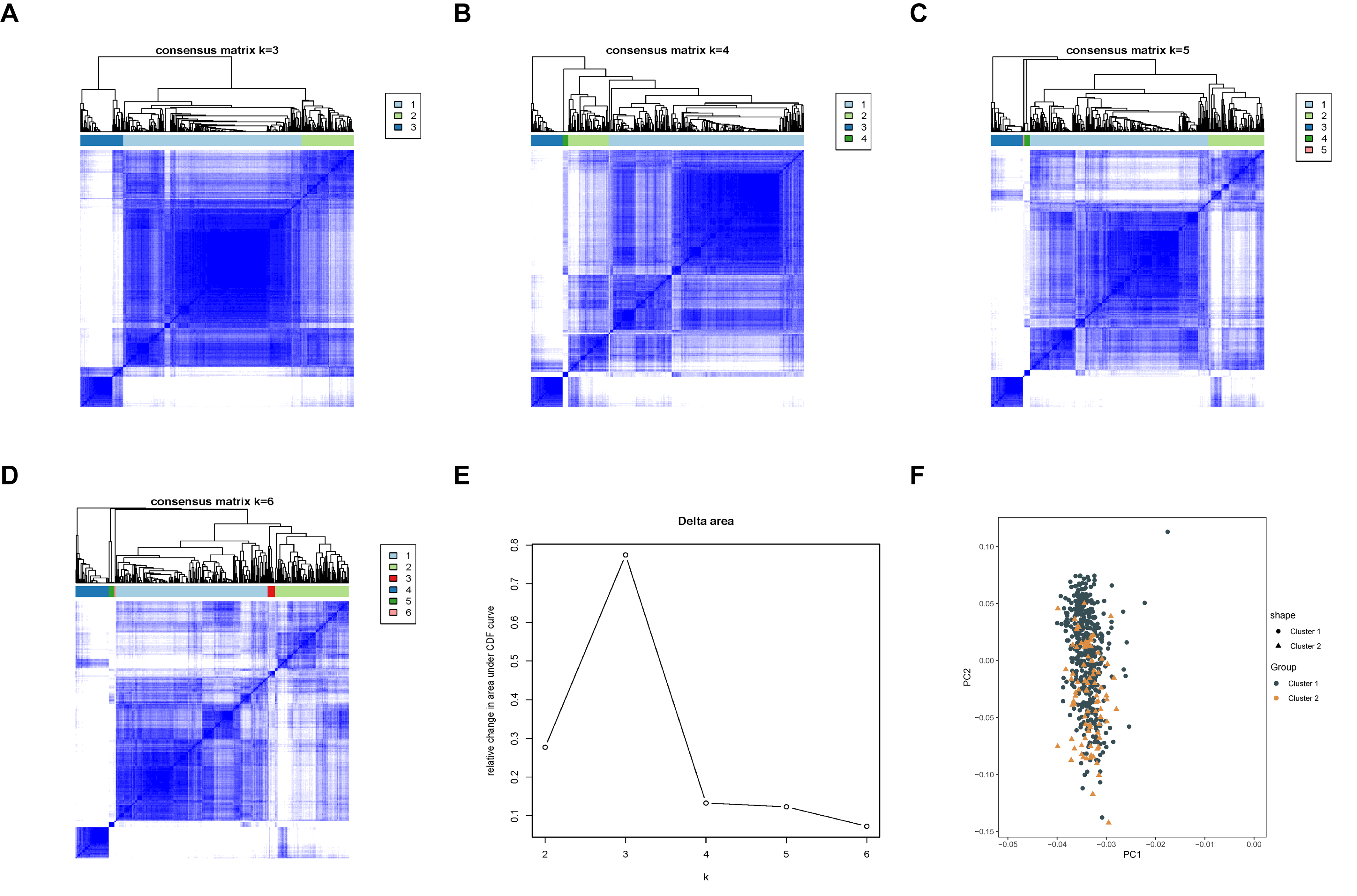

Supplement: Supplementary Figure 1 — Consensus clustering for ferroptosis regulators in clear cell renal cell carcinoma (ccRCC). (A–D) Four heat maps exhibit the clustering matrix for ferroptosis regulators in ccRCC patients for k = 3, 4, 5, and 6. The tighter and clearer the clusters are, the more optimal the cluster. (E) Delta area curve of consensus clustering for k = 2–6. (F) Principal component analysis of ccRCC patients' ferroptosis regulator expression profiles demonstrates two patient clusters. [file Image_1.TIF]
