# Supplementary material for: Comprehensive Analysis of Ferroptosis Regulators With Regard to PD-L1 and Immune Infiltration in Clear Cell Renal Cell Carcinoma
Source: Front Cell Dev Biol. 2021 Jul 5;9:676142. doi: 10.3389/fcell.2021.676142 (PMC8287329; doi:10.3389/fcell.2021.676142)
Supplement: Supplementary Table 1 — The abbreviations of multiple cancer types, ferroptosis regulators and immune-related checkpoints in this study. [file Table_1.DOCX]

| Supplementary Table 1. The abbreviations of multiple cancer types, ferroptosis regulators and immune-related checkpoints in this study. | | | |
| --- | --- | --- | --- |
| Abbreviation | Full name |  |  |
| ACC | Adrenocortical carcinoma | | |
| BLCA | Bladder Urothelial Carcinoma | | |
| BRCA | Breast invasive carcinoma | | |
| CESC | Cervical squamous cell carcinoma and endocervical adenocarcinoma | | |
| CHOL | Cholangiocarcinoma | | |
| COAD | Colon adenocarcinoma | | |
| DLBC | Lymphoid Neoplasm Diffuse Large B-cell Lymphoma | | |
| ESCA | Esophageal carcinoma | | |
| GBM | Glioblastoma multiforme | | |
| HNSC | Head and Neck squamous cell carcinoma | | |
| KICH | Kidney Chromophobe | | |
| KIRC | Kidney renal clear cell carcinoma | | |
| KIRP | Kidney renal papillary cell carcinoma | | |
| LAML | Acute Myeloid Leukemia | | |
| LGG | Brain Lower Grade Glioma | | |
| LIHC | Liver hepatocellular carcinoma | | |
| LUAD | Lung adenocarcinoma | | |
| LUSC | Lung squamous cell carcinoma | | |
| MESO | Mesothelioma | | |
| OV | Ovarian serous cystadenocarcinoma | | |
| PAAD | Pancreatic adenocarcinoma | | |
| PCPG | Pheochromocytoma and Paraganglioma | | |
| PRAD | Prostate adenocarcinoma | | |
| READ | Rectum adenocarcinoma | | |
| SARC | Sarcoma | | |
| SKCM | Skin Cutaneous Melanoma | | |
| STAD | Stomach adenocarcinoma | | |
| TGCT | Testicular Germ Cell Tumors | | |
| THCA | Thyroid carcinoma | | |
| THYM | Thymoma | | |
| UCEC | Uterine Corpus Endometrial Carcinoma | | |
| UCS | Uterine Carcinosarcoma | | |
| UVM | Uveal Melanoma | | |
| ACSL4 | acyl-CoA synthetase long-chain family member 4 | | |
| ALOX15 | arachidonate 15-lipoxygenase | | |
| ATP5G3 | ATP synthase, H+ transporting, mitochondrial Fo complex subunit C3 | | |
| CARS | cysteinyl tRNA synthetase | | |
| CS | citrate synthase | | |
| DPP4 | dipeptidyl-dippeptidase-4 | | |
| GLS2 | glutaminase 2 | | |
| PCAT3 | lysophosphatidylcholine acyltransferase 3 | | |
| NCOA4 | nuclear receptor coactivator 4 | | |
| RPL8 | ribosomal protein L8 | | |
| TFRC | transferrin receptor | | |
| SAT1 | spermidine/spermine N1-acetyltransferase 1 | | |
| SLC1A5 | solute carrier family 1 Member 5 | | |
| FDFT1 | farnesyl-diphosphate farnesyltransferase 1 | | |
| CISD1 | CDGSH iron sulfur domain 1 | | |
| FANCD2 | Fanconi anemia complementation group D2 | | |
| GPX4 | glutathione peroxidase 4 | | |
| HSPB1 | heat shock protein beta 1 | | |
| MT1G | metallothionein-1G | | |
| NFE2L2 | nuclear factor, erythroid 2 like 2 | | |
| SLC7A11 | solute carrier family 7 member 11 | | |
| EMC2 | ER membrane protein complex subunit 2 | | |
| HSPA5 | heat shock protein family A member 5 | | |
| CDKN1A | cyclin-dependent kinase inhibitor 1 | | |
| CD274/PD-L1 | cluster of differentiation 274/Programmed cell death 1 ligand 1 | | |
| CTLA4 | cytotoxic T-lymphocyte antigen 4 | | |
| LAG3 | lymphocyte-activation gene 3 | | |
| PDCD1LG2 | Recombinant Programmed Cell Death Protein 1 Ligand 2 | | |
| SIGLEC15 | sialic acid binding Ig-like lectin 15 | | |
| IDO1 | indoleamine 2,3-dioxygenase 1 | | |
| PDCD1 | programmed cell death 1 | | |
| TIM-3 | T cell immunoglobulin domain and mucin domain-3 | | |
