# Supplementary material for: Comprehensive Analysis of Ferroptosis Regulators With Regard to PD-L1 and Immune Infiltration in Clear Cell Renal Cell Carcinoma
Source: Front Cell Dev Biol. 2021 Jul 5;9:676142. doi: 10.3389/fcell.2021.676142 (PMC8287329; doi:10.3389/fcell.2021.676142)
Supplement: Supplementary Table 2 — The patients' clinical information (n = 22) in this study. [file Table_2.DOCX]

| **Supplementary Table 2. The patients’ clinical information(n=22) in this study.** | | | | | | |
| --- | --- | --- | --- | --- | --- | --- |
| **No.** | **Age** | **Sex Pathological diagnosis** | | **pT Stage** | **Fuhrman Grade** | |
| 1 | 65 | male | ccRCC | T1b | I |  |
| 2 | 51 | female | ccRCC | T1b | II |  |
| 3 | 67 | male | ccRCC | T2a | I |  |
| 4 | 53 | female | ccRCC | T1b | I |  |
| 5 | 56 | male | ccRCC | T2a | III |  |
| 6 | 57 | male | ccRCC | T1a | I |  |
| 7 | 54 | male | ccRCC | T3a | II |  |
| 8 | 65 | female | ccRCC | T1b | I |  |
| 9 | 63 | female | ccRCC | T1b | II |  |
| 10 | 60 | male | ccRCC | T4 | II |  |
| 11 | 54 | female | ccRCC | T3a | I |  |
| 12 | 58 | female | ccRCC | T1b | I |  |
| 13 | 59 | male | ccRCC | T3b | III |  |
| 14 | 43 | male | ccRCC | T1a | I |  |
| 15 | 75 | male | ccRCC | T1a | I |  |
| 16 | 62 | male | ccRCC | T1b | II |  |
| 17 | 74 | male | ccRCC | T1b | I |  |
| 18 | 68 | female | ccRCC | T3a | II |  |
| 19 | 59 | female | ccRCC | T1b | I |  |
| 20 | 55 | male | ccRCC | T3a | II |  |
| 22 | 49 | female | ccRCC | T2a | I |  |
| 22 | 55 | female | ccRCC | T1b | II |  |
