# Supplementary material for: Comprehensive Analysis of Ferroptosis Regulators With Regard to PD-L1 and Immune Infiltration in Clear Cell Renal Cell Carcinoma
Source: Front Cell Dev Biol. 2021 Jul 5;9:676142. doi: 10.3389/fcell.2021.676142 (PMC8287329; doi:10.3389/fcell.2021.676142)
Supplement: Supplementary Table 3 — The oligonucleotides used in this study. *F, forward primer; R, reverse primer. [file Table_3.DOCX]

| **Supplementary Table 3. The oligonucleotides used in this study.** | | | |  |
| --- | --- | --- | --- | --- |
| Name^*^ |  | Sequence (5’->3’) | |  |
| GAPDH | Forward primer | ACAACTTTGGTATCGTGGAAGG | | |
|  | Reverse primer | GCCATCACGCCACAGTTTC | | |
| CARS | Forward primer | CCATGCAGACTCCACCTTTAC | | |
|  | Reverse primer | GCAATACCACGTCACCTTTTTC | | |
| * F, forward primer; R, reverse primer. | | | |  |
|  |  |  |  |  |
